# Do the interpersonal effects of gamified online destination websites better stimulate tourists’ travel intentions?

**DOI:** 10.1371/journal.pone.0331397

**Published:** 2025-10-06

**Authors:** Hengji Wang, Mingze Qu, Yang Liu, Wenbo Li

**Affiliations:** 1 School of Art and Design, Zhejiang Sci-Tech University, Hangzhou, China; 2 Northumbria School of Design, Northumbria University, Newcastle, The United Kingdom; Farhangian Teacher Education University: Farhangian University, IRAN, ISLAMIC REPUBLIC OF

## Abstract

With the growing prevalence of user-generated content in disseminating travel information, the current design of online destination websites should emphasize interactive engagement and fulfill users’ increasing social needs, thereby aligning with the behavioral characteristics and development trends of the digital age. This study conducts two empirical studies to examine the feasibility of integrating gamification into destination platforms and its influence on interpersonal value within users’ decision-making processes. Through scenario-based simulations comparing feedback from 931 participants on gamified versus non-gamified websites, the study applies structural equation modeling to test path coefficients and confirm the effectiveness and advantages of gamification, as well as the role of interpersonal value in shaping decision-making pathways. This study found that: (1) Gamification effectively brings personal hedonic and utilitarian value, is suitable for destination websites, and positively influences user decisions. (2) There is no significant difference between gamified and non-gamified experiences in terms of utilitarian and hedonic value experiences, nor in the influence of utilitarian value on decision-making. (3) The personal value experience brought by gamification will positively influence users’ interpersonal value. (4) Interpersonal value—experience sharing, identity recognition, and social support—under gamified experiences will positively influence user decisions. These findings reveal that gamified design can not only effectively enhance the practical attributes of online destination websites but also significantly strengthen individuals’ information dissemination about the destination and activate the website’s interpersonal effects. This makes gamification a new model for optimizing the information display of existing online destination websites, and it highlights the crucial role of interpersonal value in studying consumer decision-making paths.

## 1. Introduction

Tourism is recognized as one of the largest and fastest-growing industries globally [1]. While tourism creates numerous opportunities for consumers, it also brings significant decision-making dilemmas, such as “travel decision paralysis”—where tourists’ decision efficiency decreases when there are too many destination options [2]. Therefore, optimizing the pre-trip experience has become a critical factor influencing tourist decisions [3]. Destination Marketing Organizations (DMOs) typically convey pre-trip experiences indirectly through promotional materials such as websites, brochures, travel guides, and word-of-mouth recommendations [4,5]. However, as the tourism industry deeply integrates into the information age [6], its highly digitized and e-commerce-driven characteristics make online platforms the primary source for tourists to obtain information and make decisions [7–9]. These websites often combine functional and aesthetic elements, attempting to evoke users’ emotional experiences [10]. Online Destination Experiences (ODEs) typically stimulate consumers’ subjective evaluations of the hedonic and utilitarian values conveyed by the website. Although this value perception varies among individuals [11], a general commonality in their design can still be observed across a large number of online travel websites in terms of stimulating these two types of values [12,13].

With the transition from Web 1.0 to Web 2.0 [14], a new “Travel 2.0” paradigm emerged, characterized by user-generated content (UGC) and real-time interaction [15]. Social media platforms and virtual communities provide tourists with diverse information channels [16,17], enabling them to share opinions and travel experiences. As a result, information is no longer solely controlled by tourism enterprises, and users can disseminate and share information through free and powerful tools such as blogs, social networks, and virtual communities [15–17], collectively known as Travel 2.0 applications. These social attributes form a complex dimension that influences users’ pre-trip decision-making processes [18,19]. For instance, when users see their friends, family, or online influencers visiting a destination on social media, they may be more inclined to make a travel decision [20].However, despite the important role interpersonal value plays in influencing users’ travel decisions, information from unofficial channels is often insufficient to serve as the core basis for final decision-making. In contrast, users tend to trust publicly disclosed content from official platforms [12,21]. some official institutions, while ensuring their own credibility, also utilize social media or platforms to disseminate information. Furthermore, destinations that rapidly gain popularity on social media often enhance their credibility and word-of-mouth effect through the intervention of official institutions. [22], thereby stimulating tourists’ actual intention to visit.

Online destination websites integrate information and communication technologies to offer highly interactive, stimulus-driven guided experiences [23], and include detailed information and interactive functionalities [24]. These websites also incorporate multimedia elements, such as high-definition images, videos, and virtual tours. However, online destination websites primarily focus on pre-trip planning, and there is limited literature regarding how these sites can stimulate consumers’ interpersonal value experiences [12]. This phenomenon highlights the need for further exploration into how the experiential mechanisms of interpersonal value can be effectively integrated into the design of official destination websites. Such integration would enhance the transmissibility and influence of the website, ultimately expanding the destination’s overall perception and attractiveness, while also addressing the current research and application gap concerning the propagation of interpersonal value and decision-making influence on official platforms.

Gamification design, as an emerging approach within mature technological development [25], serves as a mechanism to enhance user experience. It supports tourists during the pre-trip website browsing phase and provides an immersive content framework that integrates website information. Moreover, by enhancing engagement with user-generated content, gamification fosters interpersonal interactions, influencing users’ perceptions of a destination’ s reputation [26]. Although research indicates that gamification design in the tourism industry remains limited [27], several studies suggest that gamification can enhance hedonic attributes, user experience, and perceived benefits. This indicates that gamification could be a suitable strategy to enhance ODEs. However, no existing research has verified whether gamified destination websites can positively influence tourists’ hedonic and utilitarian values, leading to pre-trip decisions. This study aims to bridge this research gap while also investigating whether interpersonal relationships, as previously discussed, are activated within gamification mechanisms to influence decision-making.

This study aims to explore the applicability of gamification in online tourism destination website design and its effectiveness in enhancing user experience, further analyzing the role of interpersonal value in user decision-making during human-computer interaction. The core research questions include: How to verify the adaptability and advantages between gamification and destination websites? How to confirm the existence of interpersonal value in gamified websites and further verify its association with user decision-making? To answer these questions, this study employs a mixed experimental design. First, a control experiment (gamified group vs. non-gamified group) is designed to verify preliminary hypotheses regarding technological adaptability and experience enhancement effects. Subsequently, Structural Equation Modeling (SEM) will be used to analyze the influence of hedonic, utilitarian, and interpersonal value factors, generated by tourists in a gamified environment, on their decision-making, thereby exploring new possibilities for Online Destination Experiences (ODEs). The experimental scenario simulates tourists’ actual online information search process, and strict control over covariates such as information density and multimedia composition is maintained to ensure experimental validity.

## 2. Literature review and theoretical background

### 2.1 ODEs and user travel decision-making

Existing theoretical frameworks have developed a comprehensive analysis of the content architecture of ODEs, defining their core information modalities are defined as visual symbols, narrative texts, and dynamic images [7]. Some online destination websites integrate unique local experiences—such as Hawaiian-style beach layouts or interactive features specific to scenic areas—which collectively shape tourists’ pre-travel experiences [12]. From the perspective of the effectiveness path of ODEs, sensory, intellectual, emotional, and behavioral stimuli continuously shape tourists’ intersubjective perception of destination authenticity [28–30], thereby deeply engaging in the intention formation process of tourism consumption willingness and the preliminary evaluation of scenic spots [31,32]. However, this approach emphasizes post-travel revisit surveys and fails to specifically deconstruct the behavioral impact of online experiences on the pre-decision stage. From the perspective of ODEs’ impact on decision-making, this consumer behavior predates the rise of the virtual economy [33].

Through empirical research in e-commerce contexts, customers autonomously evaluate a product based on aspects such as “web atmosphere,” “visual design,” “product categorization,” “after-sales service,” or “information quality,” thereby seeking two types of benefits [34]. These are the individual values of hedonism (aesthetic, experiential, and entertainment-related benefits) and utilitarianism (functional, instrumental, and practical benefits), which jointly play a coordinating role in purchase decisions [35–38]. Scholars have collected data on users’ online shopping behavior and found that even though each consumer has different activation points, their purchase motivation is still influenced by website design content [39]. Köchling et al. applied this paradigm to the study of tourism digital interfaces, proposing that ODEs will effectively stimulate tourists’ hedonic and utilitarian values, and this value experience will influence tourists’ pre-trip decisions [12]. However, because their experiments were conducted only on existing websites, lacking exploration of possibilities brought by specially designed mechanisms, the explanatory power of this model remains limited.

Hirschman et al. proposed the concept of hedonic motivation from different perceptions: it refers to consumer behaviors that seek pleasure, fantasy, arousal, sensory experience, and enjoyment [11]. That is to say, hedonic value should belong to the consumer’s personal value. Hedonic consumers like shopping because they enjoy the shopping process itself [39]. Even though each consumer has different evaluation standards for hedonic experience, such as considering their financial situation and consumption standards [40], planned designs and activities will serve as a hedonic motivation to mobilize consumer decisions [41]. This motivation is not merely stimulated by single novelty, pleasure, or interesting design elements, but is the result of multiple stimuli acting in concert. Furthermore, the impact of a single stimulus gradually diminishes over time [42]. Therefore, in Köchling et al.’s research, they believe that personal hedonic experience is a high-level psychological representation experience, which is comprehensively influenced by users’ personal emotions, senses, and the passage of time [11,12]. However, when designing Online Destination Experiences (ODEs), stimulating consumers’ hedonic motivation is not difficult to achieve. Huang et al. combined tourism with virtual reality (VR), and the flow experience significantly extended consumers’ expectations for future travel experiences [43]. This experience not only reflects sensory and emotional elements but also affects consumers’ long-term emotional trajectories [44]. Additionally, it strengthens the connection between consumers and the product, promoting continuous interaction through generated emotions [45]. Affective forecasting indicates that hedonic value significantly influences consumer decisions [46–49]. Furthermore, the long-term hedonic value generated by consumers will foster an emotional bond with the product, thereby enhancing consumer loyalty and stickiness [34,45,50].

Consumers with hedonic motivation pay closer attention to the atmosphere conveyed by online information, while consumers with utilitarian motivation focus more on the efficiency attributes of online information content. These two dimensions are not mutually exclusive [34]. Personal utilitarian value is goal-oriented [45,51], such as purchasing goods to satisfy specific needs [41,52]. In ODEs, intelligence, social interaction, and behavior will serve as evaluation dimensions for the excitation of personal utilitarian value. They symbolize whether users can learn relevant destination information from the website and provide basic support for their travel plans, including but not limited to understanding transportation, destination location, local customs, and so on [12]. Therefore, in Maslow’s hierarchy of needs and priority principles, utilitarian value is defined as a more fundamental dimension than hedonic value. A product’s ability to stimulate consumers’ utilitarian value often relies on its most basic functions, existing as a necessity, whereas factors that stimulate hedonic value are emotional luxuries [36,53,54]. From a consumer’s perspective, utilitarian value is often more practical than hedonic value until their minimum expectations for preventive goals are met [36]. Therefore, although enhancing consumers’ hedonic value can boost word-of-mouth and repurchase intentions, if consumers’ utilitarian value is not satisfied, they may experience negative emotions or even anger [44].

### 2.2 Gamification

Gamification is an incentive technique that applies game mechanisms such as rewards and challenges to non-game contexts, thereby eliciting positive behavioral outcomes [55]. According to Simões et al., gamification has been integrated into websites, business services, online communities, and marketing campaigns to encourage user engagement [56–58]. This mechanism serves as a new marketing approach for online platforms, effectively conveying cognitive content to educate consumers and inspire them to engage more deeply with the platform experience [59]. Companies such as Recyclebank, McDonald’ s, Pepsi, Samsung, and Nike have evaluated the use of gamification in marketing communication. Additionally, various popular websites, including Twitter, eBay, Facebook, and Foursquare, have incorporated game elements to encourage greater user participation.

Research indicates that gamification can effectively stimulate users’ functional behaviors and mobilize their emotions [45]. This means it encompasses several perceived attributes, such as aesthetics, usability, ease of use, trustworthiness, and interactivity, characteristics that align with the needs of online destination platforms [60–62]. Furthermore, gamification possesses social attributes, allowing consumers to build interpersonal connections by experiencing hedonic and utilitarian aspects within the game. For example, helping others achieve utilitarian goals in a game can satisfy individual personal values, thereby fostering the formation of common interest circles through game design [63]. Additionally, leveraging social interaction to influence user decisions has proven effective [64]. In terms of content dissemination and user experience, gamification can be categorized into content gamification (applying game elements to software content and integrating game-like content) and structural gamification (motivating and engaging users through rewards, such as points, badges, or achievement-based special actions) [65].

Despite existing research having explored the impact of gamification on tourism consumers and demonstrated that its interactivity and entertainment can effectively stimulate personal hedonic and utilitarian motivations. [66,67], the role of unique gamification mechanisms in stimulating consumer social interaction and interpersonal relationships, which may affect consumer decision-making processes, still needs to be considered. Based on this, the introduction of gamification has the potential to alter the evaluation criteria for online destination platform experiences. Moreover, while gamification is regarded as a future trend in marketing and is believed to enhance product experience and user engagement [68,69], most gamification mechanisms are currently primarily applied in the educational domain, with relatively limited penetration in the tourism industry [27]. Nevertheless, gamification has shown its potential in areas such as tourism promotion, digital tourism, and sustainable environmental development [70–72]. In conclusion, the advantages of gamification are closely related to the core interests of online destination platforms, which is also our main motivation for designing a structured gamified online destination website and exploring whether gamification can enhance the overall experience effect of such platforms.

### 2.3 The impact of gamification-designed destination websites on travel decision-making

A gamification-based design encompasses both hedonic and utilitarian characteristics. The self-determination theory can explain the sources of players’ hedonic experiences, wherein player motivation is generally classified into two types: intrinsic motivation and extrinsic motivation. The study by Simões et al analyzed tourists’ motivations for engaging in gamified travel using the Q methodology and [56], based on this, categorized tourist types, and concluded that intrinsic motivation pertains to the satisfaction or pleasure derived from playing games [73,74], because according to the flow experience induced by gamification, players engage with game content, overcome various challenges, and thereby experience positive emotions such as surprise, a sense of achievement, excitement and fun, curiosity, and enjoyment [75], and go on to achieve the predefined outcomes set by designers, or even exceed their original plans [76], while extrinsic motivation occurs when individuals expect to receive known external rewards, including tangible rewards (such as prizes or financial incentives) and psychological rewards (such as praise or encouragement) [77].

The selection of gamification elements also drives users to develop such motivation, these elements include [65]: a. Story, b. Clear Goals, c. Challenge, d. Time Limit, e. Progression, f. Reward. These elements serve utilitarian functions, incorporating mechanisms designed to help users acquire knowledge and achieve educational objectives [78], even influencing users’ perceptions and attitudes [72,79], for instance, in the study by [80], gamification was utilized to facilitate users’ effective learning of destination-related knowledge and promote environmental awareness.

Additionally, gamification can enhance players’ trust and loyalty towards a brand [67]. Moreover, gamification also possesses social attributes, capable of creating social value through interactions such as appreciation, praise, and mutual exchange, thereby fostering a sense of community, building social connections, and promoting future interactions (with the brand and other consumers) [81,82]. It can also generate utilitarian ODEs designs, which involve rational thinking and require a higher level of engagement compared to ODEs designs with hedonic motivations [83]. However, although existing research has not yet attempted to integrate gamification into online destination platform design, based on the aforementioned research findings, we can infer that integrating gamification into online destination platforms will help achieve key content elements such as website effectiveness, efficiency, aesthetics, trust, interactivity, and perceived experience, while introducing attractive experiences, thereby enhancing both hedonic and utilitarian experiences. Therefore, this study proposes the following basic propositions:

H1: Gamified destination platforms positively influence consumers’ hedonic value experience on the website.

H2: Compared to non-gamified websites, gamified destination platforms have a more significant positive influence on consumers’ hedonic value experience.

H3: Gamified destination platforms positively influence consumers’ utilitarian value experience on the website.

H4: Compared to non-gamified websites, gamified destination platforms have a more significant positive influence on consumers’ utilitarian value experience.

The anticipation phase of travel is closely related to the happiness derived from travel. An empirical study by Wei indicates that the travel behavior cycle can be divided into five major stages: the pre-contemplation phase, the planning contemplation phase [84], the action preparation phase, the travel phase, and the maintenance phase. The first three stages all occur within the process of constructing travel anticipation before actual departure. Notably, these three stages are closely related to tourists’ overall anticipated happiness from travel. The traditional choice set model abstracts tourism decision-making as a staged filtering process driven by information search, consisting of three core dimensions: action set, evoked set, and travel propensity [85,86]. It effectively dissects the decision-making process into seven steps [87]: 1. Identifying decision-making needs. 2. Defining goals. 3. Constructing the choice set. 4. Making a selection from alternatives. 5. Purchasing and/or consuming the product/service. 6. Post-purchase evaluation. However, certain tourists/users may skip some decision-making stages if they exhibit the following characteristics: brand loyalty, familiarity with the product/service due to past experience, influence from social factors, high engagement in the decision-making process, or routinization of the decision-making process [19]. The study also clearly summarizes that travel decision-making consists of internal constraints, interpersonal constraints, and structural constraints [88], which are closely related to the gamification aspects discussed earlier.

ODEs with hedonic attributes focus on emotional pleasure and entertainment acquisition [41], while those with utilitarian attributes emphasize information acquisition efficiency and rational economic evaluation [67,89], for example, the economic perception of “value for money.” Consumers are influenced by these elements, which alter their shopping preferences and profoundly impact their final decision trajectory through intentional conversion mechanisms [90]. According to Maser and Weiermair, “in the tourism industry, information is considered one of the most important factors influencing and determining consumer behavior” [91,92].

From a cognitive perspective, a detailed examination of destination decision-making reveals that before tourists become familiar with a destination, they experience a high degree of uncertainty regarding its image. For instance, they may be uncertain about transportation conditions, scenery, and visitor flow at the destination. Tourists simulate consumption scenarios and anticipate the consequences of their experiences, forming cognitive and emotional foundations that influence their travel decisions [93].In other words, if tourists lack cognitive information about a destination, their willingness to visit may become unclear due to the many uncertainties associated with the trip [94]. Gamification mechanisms leverage players’ interactive and dynamic cognitive processes to trigger emotions and create static cognitive outcomes that reflect their understanding.Therefore, this study proposes the following hypothesis:

H5: Gamified destination experiences will stimulate consumers’ utilitarian value, thereby positively influencing their travel decisions.

H6: Compared to non-gamified website experiences, gamified destination experiences will more positively stimulate consumers’ utilitarian value, thereby influencing their travel decisions.

Consumers may make choices based on their mental representations of products. In tourism applications, authenticity and a sense of sincerity are crucial means of achieving this goal. The process by which consumers select a destination may be intuitive, emotional, or implicit rather than purely cognitive [95–97]. From this perspective, emotions function similarly to fundamental cognitive processes, representing value judgments [98]. Relevant information and elements provided by websites can stimulate tourists’ emotions, thereby enhancing their satisfaction, purchase intentions, and actual purchase behavior. Consequently, consumers’ attitudes toward the products or services offered by a company depend on whether their utilitarian or hedonic expectations are met in their online experience [45]. This study will explore whether hedonic value experiences serve as a mediating factor that influences tourists’ willingness to visit a destination. Therefore, the following hypotheses are proposed:

H7: Gamified destination experiences will stimulate consumers’ hedonic value, thereby positively influencing their travel decisions.

H8: Compared to non-gamified website experiences, gamified destination experiences will more positively stimulate consumers’ hedonic value, thereby influencing their travel decisions.

### 2.4 The impact of gamification-based interpersonal value on user decision-making

Interpersonal Value is defined as the benefits individuals gain in terms of emotional support, social recognition, and information exchange through the process of interactions and engagements with others [99–101]. This value reflects the impact of interpersonal relationships on psychological satisfaction, social status, and information acquisition [102]. In the field of tourism research, interpersonal value has been recognized as a crucial factor influencing tourists’ decision-making [69]. In their study [103], referred to interpersonal influence as word-of-mouth (WOM), emphasizing its role in shaping user decisions. WOM, in turn, is significantly affected by interpersonal interactions within virtual communities, websites, blogs, and other online platforms. Simply put, before traveling, tourists often interact with friends, family, or online communities to obtain destination-related information as a form of advice and support. From the perspective of the gaming domain, social influence in the Unified Theory of Acceptance and Use of Technology is considered a key factor driving the adoption of gamification [104]. This concept refers to how others’ perceptions influence an individual’ s decision to adopt a particular technology, highlighting whether users choose to embrace it [105]. Eisingerich found that if most friends or peers engage with a certain game [106], individuals are likely to follow suit. Similarly, gamification can become a motivational driver for interpersonal value [107], fostering social interactions with friends or local residents. Moreover, the presence of gamification also influences destination-related WOM information [26]. Consequently, the value provided by these destination gaming platforms significantly impacts users’ interpersonal value, ultimately shaping their destination choices. Based on these insights, this study establishes the following fundamental judgments:

H9: Gamified destination platforms will positively stimulate tourists’ interpersonal value experience.

Additionally, the interpersonal value generated by gamification guides potential users unfamiliar with the game to learn about and experience it. From the perspective of social influence, the transition from a potential user group to an engaged user group undergoes three transformation processes—experience sharing, identity recognition, and social support [108–110]. Furthermore, by popularizing and promoting the destination, existing users can influence their family and friends, expanding the social impact of destination-related information [111]. The level of familiarity with the destination also becomes shareable content, garnering support from family, friends, and social circles. This, in turn, enhances the hedonic experience provided by gamification [112].

As discussed above, the interpersonal value generated by gamification leverages hedonic and utilitarian experiences to enable users to better understand informational content. Thus, we propose the following hypotheses:

H10: Utilitarian experiences driven by gamified design prompt consumers to share destination information, thereby positively influencing tourists’ interpersonal value experience.

H11: Hedonic experiences driven by gamified design prompt consumers to share destination information, thereby positively influencing tourists’ interpersonal value experience.

Gamification can enhance player loyalty, and the communities established based on this loyalty may influence potential game users—those who are not interested in or have never experienced this type of game—to become engaged users who enjoy the game and understand its content [113]. Simply put, the rewards and fun mechanisms inherent in gamification design can serve as factors attracting tourists to visit destinations.

Interpersonal value is also closely related to travel decision-making. The development of Web 2.0 has demonstrated that social media influences the online image of destinations through WOM, thereby affecting tourist decision-making [7,114]. Friends, family, and relatives are influenced by destination images disseminated via social media, with various users—including product developers, influencers, and acquaintances—playing a role in this dissemination. During social interactions, people tend to listen to multiple suggestions before making decisions. Compared to other sources of information, the opinions of friends and family are often considered more credible, and users may even fear that opposition from friends could deter them from proceeding with their decision [19,115].

Although there is extensive literature on how social media influences destination-related decisions, fewer studies have examined how players disseminate information within their social circles from a gamification perspective. Based on the above discussion, we propose the following hypothesis:

H12: The interpersonal value experience that gamified destination experiences bring to consumers will positively influence their travel decisions.

## 3. Methodology

### 3.1 Research models

This study establishes two progressive models: The first model verifies the efficiency-enhancing effect of gamification design on tourist decision-making and its compatibility with online platforms. A comparative experiment confirms that gamified websites stimulate travel intentions more effectively than traditional platforms. Based on this conclusion, the second model extends beyond the original hedonic and utilitarian dimensions by introducing the dimension of interpersonal interaction value. It systematically examines the mediating pathways and impact intensity of the three value elements in the decision-making chain. This study integrates both applied validation and theoretical innovation: it not only demonstrates the effectiveness of gamification as a platform optimization strategy but also reveals its unique mechanism, distinct from traditional experience evaluation frameworks, by deriving new driving forces such as interpersonal relationships. This dual verification confirms both the value of technological applications and expands the explanatory dimension of user experience theory.

#### 3.1.1 Model 1: The impact of gamification-designed destination websites on travel decision-making.

**Fig 1:**
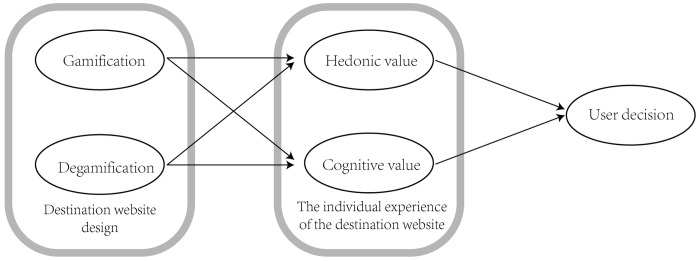
Experiment 1 Model: The influence of gamified design of destination website on travel decision making.

Model 1 focuses on gamification as a new approach to platform website design, exploring the decision-making differences between gamified websites and non-gamified destination websites. Therefore, in this section, we should examine whether gamification can be integrated with destination platforms and use a literature review and experiments to demonstrate the feasibility of gamification in influencing decision-making paths. Subsequent experiments will validate its feasibility and the correctness of our hypothesis through a comparative group method. Based on this, we propose the research model in (Fig 1).

#### 3.1.2 Model 2: The impact of gamification-based interpersonal value on user decision-making.

**Fig 2:**
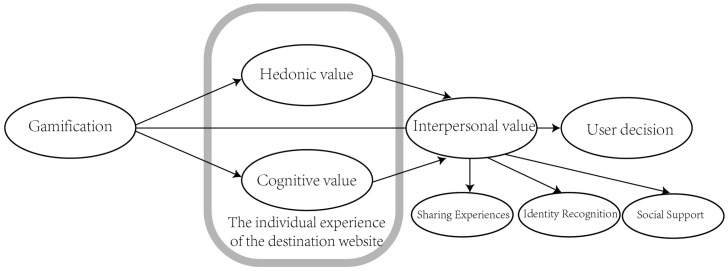
Experiment 2 Model: The influence of gamified interpersonal value on user decision making.

In this section, we will explore additional factors influencing user decision-making after the optimization of destination platforms through gamification, based on the results of Model 1: interpersonal value. Through a literature review and empirical analysis, we will demonstrate that the interpersonal value generated by gamification is supported by objective evidence. We will also establish the relationship between interpersonal value and other existing factors, as well as its ultimate impact on user decision-making. Consequently, we have developed the following model framework (Fig 2).

### 3.2 Website design

This study sets up two types of online tourism platform interfaces: (1). a conventional multimedia information platform (without gamification design). (2). a gamified interactive platform (integrating game mechanics into multimedia presentation). Both maintain consistency in landscape presentation, informational content, and visual elements; the primary distinction lies in the dimension of interactive architecture design. Based on gamification design paradigms, the experimental group’ s platform additionally integrates the following validated interactive elements: goal-challenge systems, instant achievement rewards, and dynamic feedback mechanisms [116,117]. Meanwhile, the control group adopts a standard tourism website framework. During the design process, we ensure consistency in destination-related content between the gamified and standard websites. However, additional visual elements (e.g., reward screens) will be introduced exclusively in the gamified platform to meet design requirements.

In implementing destination gamification, various approaches can be adopted, such as luck-based mechanisms (e.g., lotteries, dice games, and other random incentive mechanisms)or knowledge-based quizzes [118,119], where players must continuously improve their abilities through repeated testing to complete the game [80]. This study selects quiz-based gaming as the core interaction method, as it compels users to process destination information in depth, thereby achieving an organic integration of educational objectives and gaming experiences. In selecting gamification elements, this study incorporates avatars (role-playing), challenges (quizzes), real-time feedback, progress tracking, storytelling, clear goals, and rewards as the main elements. Further details on implementation will be elaborated in subsequent sections.

The experimental platform is set in Gongshu District, Hangzhou, based on the following considerations: as a demonstration area for digital economy development in the Yangtze River Delta urban agglomeration, (1) it has a well-established tourism infrastructure and emerging attraction clusters; (2) online tourism service platforms remain in an underdeveloped stage; and (3) the city is one of China’s most advanced hubs for internet enterprises [120]. Users in this area have already formed a stable behavior pattern of obtaining tourism information via social media platforms (WeChat/Weibo/Little Red Book). The platform design thus integrates official government data resources to construct core functionalities such as scenic spot reservations, ticket purchases, and route planning. This design brings many advantages to our platform, allowing us to consolidate various scenic spot information in Gongshu District as part of our content. For example, we can embed official public accounts for tourists to follow and make same-day reservations—an approach that has become common practice across China. Most websites already feature links to other ticketing services, making the integration of gamification elements an effective means to differentiate our website’ s information dissemination structure from conventional website designs. The experimental design details are available in S2-5 Fig, Due to copyright reasons, we have covered some images with copyright issues with a gray overlay.

### 3.3 Scenario-based experiment

A scenario-based experiment is a crucial research method in service marketing because it allows for easier manipulation of complex research variables while reducing research costs [121]. This method also minimizes memory bias that may arise over time with traditional recall methods and has been widely applied in recent tourism behavior studies [122]. Regarding the post-experiment questionnaire survey, primarily based on the questionnaire scales we developed, we analyzed the data by combining the questionnaires filled out by different respondents with an SEM model, thereby deriving the data content presented in the conclusions.

#### 3.3.1 Procedures.

**Fig 3:**
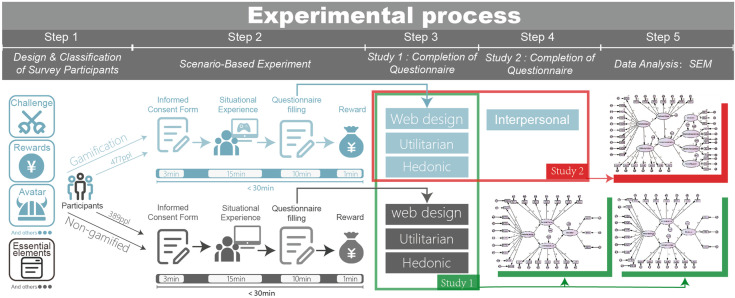
The flowchart of this experiment.

To ensure data validity, participants were required to use a designated online platform; unlimited internet searches could potentially distort the objective impression of the destination due to social media content and unexpected events, thereby interfering with questionnaire measurements [123]. After completing the scenario experiment, users needed to fill out a questionnaire to collect their experiences using the online platform and their impressions of the destination. Before the experiment began, participants received a written informed consent form stating that the experimental process was expected to take 10 to 30 minutes. The experiment would only commence after obtaining their explicit consent, ensuring they fully understood and voluntarily participated. Our overall experimental setup is shown in (Fig 3).

Finally, the following instructions were provided to both groups (gamified/non-gamified) during the experiment:

“You currently have a vacation (vacation duration depends on your travel habits). Among multiple travel options, you have chosen Gongshu District in Hangzhou. You now have 15 minutes to browse the Gongshu District online destination platform we provide. During this time, you can list your travel itinerary or learn about the destination’s transportation, accommodation, dining, scheduling, and other information. After the experiment, you will need to complete a follow-up questionnaire.”

During the experiment, participants were randomly assigned to one of two groups: one experiencing a gamified online destination platform, and the other using a non-gamified platform. Participants in the gamified group assumed the role of online customer service representatives, completing four rounds of preset scenic spot inquiry tasks (NPC inquiries, including routes, dining, transportation, etc.). Each correct answer received a gamified incentive (“accumulated bonus +100 RMB” + praise feedback) to maintain engagement [124], while incorrect answers triggered a warning interface. The ultimately generated smart itinerary could be socially shared, allowing for analysis of the impact of interpersonal communication on decisions. Finally, participants received a tangible reward (US$2). However, to prevent the reward from influencing user choices and experimental experience, participants were informed of the reward’s existence before the experiment but were not told the specific amount or form of the reward. The reward was solely compensation for their participation in this experiment and would not change based on their choices or actions during the experiment. This measure aimed to minimize potential bias induced by rewards [125], ensuring participants’ decision-making behavior reflected their true experiences and responses [126,127].

After the experiment, we provided questionnaires to both groups of participants, who were asked to complete the questionnaire content within 15 minutes. Additionally, users in the gamified group had two extra questions to verify whether their experience made them more willing to share destination information with friends. If their answer was positive (they selected neutral or above options), then our Experiment 2 questionnaire would be activated, and the software would automatically provide a second questionnaire on interpersonal value for testing, again allowing 15 minutes for completion.

Finally, we conducted a pre-survey with 120 respondents (60 for the gamified website, 60 for the non-gamified website). To ensure the reliability of the questionnaire, we tested its Cronbach’s alpha coefficient, conducted exploratory factor analysis, and performed confirmatory factor analysis to confirm the reliability of the questionnaire content and ensure the collection of valid data before the formal experiment. Ultimately, the collected data met or exceeded the standard coefficient thresholds. Although the coefficients of some items (such as HV7 and UV6, reverse-coded items) were close to the ideal threshold, considering that each condition’s sample size in the pre-survey was only 60 responses, these items will be deleted in the formal experiment, thus confirming the reasonableness of our scale development and experiment.

#### 3.3.2 Ethical consideration.

Our experiment’s recruitment method was online, with experimenters distributing experiment links in WeChat groups of Zhejiang universities (serving as official social platforms). After volunteers applied, we provided a written informed consent form, which was then distributed to volunteers by experimenters from each university. The informed consent form clearly stated the research purpose, experimental procedure, potential risks, and that participants had the right to withdraw from the study at any time without any negative consequences. All experimental data will be stored on a secure encrypted server, and participants were anonymized when filling out questionnaires, with information kept strictly confidential. We only required participants to complete relevant questionnaire questions after the experiment, ensuring the security of their information. All anonymous datasets are accessible only to the core research team, guaranteeing data confidentiality and security. This research protocol has been approved by the university and the ethics committee (Approval No.: 0227/2025). Please refer to the ethics review form in the appendix for details. The entire process strictly adhered to the ethical requirements of the Declaration of Helsinki regarding human experimentation.

### 3.4 Measurement tools and scales

#### 3.4.1 Evaluation criteria.

ODEs were evaluated using a 7-point Likert scale, ranging from “-3 = strongly disagree” to “+3 = strongly agree,” as previous research indicates that a seven-point response format performs better than five-point or eleven-point formats in this context [128]. Subsequently, all items on our questionnaire completed by participants used a 7-point Likert scale, including hedonic value, utilitarian value, interpersonal value, website evaluation, and travel decision-making.

#### 3.4.2 Scale development.

In evaluating ODEs, participants were required to assess destinations and their associated holiday experiences using multiple scales, specifically by evaluating their thoughts and feelings while Browse the website. This study is based on scales developed by Köchling and Lohmann in 2021 and 2022 [12,13], which almost cover all potential experiential aspects of ODEs. In their articles, sensory, emotional, and spatiotemporal experiences were categorized as first-order constructs within the hedonic value domain, while intellectual, social, and behavioral dimensions were categorized as first-order constructs within the utilitarian value domain. In our S1 Table scale content, hedonic value items correspond to: sensory (HV1–2), emotional (HV3–4), and spatiotemporal (HV5–6), plus one reverse-coded item for spatiotemporal (HV7). Utilitarian value items correspond to: intellectual (UV1–3), behavioral (UV4–6) where UV6 is a reverse-coded item, and social (UV7). These scale items are derived from Köchling et al.‘s research [13]. Among them, we modified intellectual items UV1 and UV3, drawing from Bae et al.’s 2022 article, as that scale better aligns with how our designed website experience mobilizes users’ intellect [129]. For the standardized evaluation dimensions of the website, we added items to Köchling’s five-dimensional item scale because our website has a certain degree of interactivity. These correspond to the items in S1 Table as follows: aesthetics (WD1–2) [12,29], usefulness (WD3–4) [12], ease of use (WD5–6) [130], trustworthiness (WD7–8) [131], and interactivity (WD9–10) [25]. Regarding the quantification of the decision questionnaire, based on Lian and Yu’s travel decision scale (DE1–2) [25], we rephrased the statements without changing their original meaning to make them easier for participants to understand. For example, the original “Really helpfulness to make travel decision” was adapted to “The website/game was very useful in my travel decision-making.” Furthermore, we removed two options: “Benefits to make travel decision” and “Easiness to make travel decision,” because our paper is not discussing the benefits or ease of decision-making, but rather focusing on the website’s influence on users’ pre-trip decisions. Therefore, we added an item regarding whether tourists would intend to visit the destination (DE3) [132].

Using only a first-order measurement model can lead to redundant item design and high correlations among items, thereby making model fit unreliable [133]. In many social science studies, latent variables often have multi-level structures, composed of multiple first-order measurement indicators and a few second-order constructs. Therefore, second-order measurement models are considered a more accurate and effective way to model complex latent variable structures [134], as they are more explanatory and concise. For our study on interpersonal value, we evaluated it from three dimensions: sharing experiences, identity recognition, and social support. These items come from different articles, so we must perform an exploratory factor analysis on this model. Simultaneously, a more cautious approach is used, which also means that in the data section we will test the R² of the second-order model to verify the explanatory power of the factors [135]. Moreover, in the data analysis, we will still establish both first-order and second-order models and compare them to verify which model is more suitable for explaining the conclusions.

Regarding the questionnaire content for Experiment 2, we referred to the development and design of social influence scales in Kang and Sedera et al.‘s research [136,137]. Since the former primarily focused on the impact of social factors on travel experience, this study made appropriate modifications based on the gamified experience. However, key items related to social value, such as internalization, compliance, and identification, were largely retained in this study. In terms of gamification, this study reinforced the role of social interaction and interpersonal value dimensions. We drew upon research by Jr, Ryan et al. to develop and design a scale measuring the impact of interpersonal value on travel decisions. Ultimately, interpersonal value was divided into three first-order constructs: experience sharing (SE1-3) [136,138], identity recognition (IR1-3), and social support (SS1-3) [137,139]. Finally, regarding the interpersonal experience questionnaire content, we added an item: “After experiencing this website, are you willing to share your travel plans or intentions with friends or family?” This item helps us assess whether the gamified website can stimulate users’ willingness to share, thereby facilitating our subsequent Research 2, which measures the website’s consideration of interpersonal value. At this stage, tourists unwilling to share will be excluded from our analysis. Ultimately, for both Experiment 1 and Experiment 2, we should perform exploratory factor analysis on the scales and final results to verify the rationality of these items.

#### 3.4.3 Data analysis.

This study utilized IBM SPSS version 27.0 and AMOS version 26.0 for statistical analysis. The data analysis procedure was as follows:

First, frequency analysis was conducted to examine demographic characteristics and exclude information that might interfere with the experiment. Reliability analysis was performed in SPSS using Cronbach’s alpha to assess the reliability of the indicators. Additionally, this study also conducted Exploratory Factor Analysis (EFA) and Confirmatory Factor Analysis (CFA) to validate the scales’ effectiveness. This step was necessary because we modified existing scales and constructed new ones by combining elements from multiple scales to ensure the reliability and validity of the final scale results. The analysis also included correlation analysis to check the proximity (i.e., correlation) between variables. Finally, Structural Equation Modeling (SEM) analysis was performed to verify the causal relationships between variables, which constitutes the core content of this study.

### 3.5 Respondent information

Our formal survey questionnaires were distributed by four trained investigators who ensured participants followed experimental guidelines and completed questionnaires during the effective post-experiment period to prevent influence on experimental results. The questionnaire was administered via the Wenjuanxing electronic platform, a survey tool developed for the Chinese context and integrated into WeChat. Volunteer recruitment took place from February 27th to March 5th, 2025. The subjects of this study were graduate students, undergraduate students, and faculty members from several universities in Zhejiang Province, China. This group was chosen because existing research indicates that students and faculty have high engagement in terms of learning ability and curiosity [140]. Additionally, the university environment covers a wide age range similar to the general social demographic structure, which makes our data collection more convenient and objective. This high level of participation and the characteristics of the group help reduce the time consumed during the experimental introduction, thereby avoiding any negative impact on participants’ enthusiasm. Experimental survey links were distributed by investigators through social platforms of various universities in Zhejiang Province; this method of experimental recruitment via social platforms is very common in Chinese universities. Considering that we conducted a total of two experiments, and the second experiment involved a second-order structural equation model, a larger sample size was used. However, respondent demographic factors were not included in the analysis. A total of 931 questionnaire responses were collected. Since prior experience with the destination might influence research results, we excluded 48 responses from users who had visited the destination more than three times. Given that we conducted a total of two experiments, with the second experiment involving a second-order structural equation model and thus a larger number of participants, we did not factor in respondent demographics. We collected a total of 931 questionnaire responses. Considering that prior experience with the destination could affect experimental results, we excluded 48 responses from users who had visited the destination more than three times. After excluding invalid questionnaires, the final number of questionnaires obtained for Experiment 1 was 389 each for the gamified and non-gamified groups (totaling 778). Based on the two initial screening questions in Experiment 1’s gamified group, 17 participants indicated unwillingness to share, and 25 participants in the gamified group had visited the destination more than three times. Subsequently, our investigators distributed an additional 105 questionnaires. Ultimately, we concluded that, as shown in the table, most respondents regularly use social media and have relatively large social circles, which will be one of the factors considered in our conclusions. After excluding some invalid questionnaires, we obtained 477 questionnaires for Experiment 2. Table 1 presents the detailed information of our screened respondents.

**Table 1:** Respondent population statistics.

	Study1		Study2	
Item	N (persons)	Share (%)	N (persons)	Share (%)
**Experience website**				
**Game**	389	50%	477	100%
**Non-game**	389	50%		
**Age**				
**Under 18 years old**	44	5.7%	26	5.5%
**18-25**	227	29.2%	144	30.2%
**26-30**	205	26.3%	126	26.4%
**31-40**	120	15.4%	71	14.9%
**41-50**	106	13.6%	58	12.2%
**51-60**	68	8.7%	42	8.8%
**Age 61 and above**	8	1.0%	10	2.1%
**The number of times the user has been to the destination**				
**0**	272	35.0%	170	35.6%
**1**	256	32.9%	160	33.5%
**2**	250	32.1%	147	30.8%
**Number of social media friends**				
**Less than 100**			41	8.6%
**101-500**			123	25.8%
**501-1000**			163	34.2%
**More than 1000**			150	31.4%
**Whether social media has become part of the user**				
**Completely disagree**				
**I don’t agree**			5	1.0%
**Slightly disagree**			4	0.8%
**Neutrality**			17	3.6%
**Somewhat agree**			66	13.8%
**More agree**			142	29.8%
**Couldn’t agree more**			138	28.9%

## 4. Results

Next, we examine the relationships between the conceptual model and hypotheses. Two experiments were conducted, both utilizing SEM in AMOS to determine the overall model fit with the data. This includes analyzing the path coefficients among latent variables in Study 1 and evaluating the feasibility of the hypotheses in Study 2.

### 4.1 Analysis of study 1 results: Path analysis of the impact of gamified and non-gamified websites on decision-making

#### 4.1.1 Reliability and validity of the measurement scale.

We employed CFA and EFA on the data to measure the reliability and validity of the scales. As shown in Table 2, the KMO measure of sampling adequacy was 0.954, significantly higher than the 0.70 threshold proposed by Hutcheson and Sofroniou [141]. Bartlett’s test of sphericity yielded a chi-square value of 12,279.602 (df = 351, p < 0.001), meeting rigorous data applicability standards [142]. These values demonstrate that our sample is sufficient and the overall model is complete. The commonalities analysis, primarily used to examine the relationship between each variable and its latent factor, showed that all items had extraction values ranging from 0.611 to 0.758. This indicates that each item has high validity and reliability in measuring the latent factor [143]. In the total variance explained, we extracted four components with initial eigenvalues greater than 1, with a cumulative explained variance of 65.347%, exceeding the recommended 50% threshold. This suggests that the extracted factors effectively represent the major variability in the data, possessing high explanatory power and validity, thus providing a more solid foundation for subsequent analysis [144]. The first component accounted for 35.325% of the initial variance, falling below the critical threshold of 50%. This indicates that multiple factors in the model collectively explain the data’s variability, reducing the risk of common method bias often associated with a single dominant factor [144].

**Table 2:** Exploratory Factor Analysis in Study 1.

Items	Component				Common Factor Variance
	1	2	3	4	
**WE1**	0.775				0.625
**WE2**	0.783				0.639
**WE3**	0.771				0.643
**WE4**	0.750				0.611
**WE5**	0.760				0.626
**WE6**	0.780				0.644
**WE7**	0.777				0.654
**WE8**	0.792				0.652
**WE9**	0.752				0.631
**WE10**	0.763				0.627
**EV1**		0.776			0.652
**EV2**		0.772			0.646
**EV3**		0.776			0.651
**EV4**		0.785			0.658
**EV5**		0.785			0.666
**EV6**		0.756			0.623
**EV7**		0.790			0.658
**UV1**			0.758		0.624
**UV2**			0.770		0.642
**UV3**			0.781		0.655
**UV4**			0.790		0.681
**UV5**			0.759		0.642
**UV6**			0.766		0.637
**UV7**			0.799		0.668
**DE1**				0.801	0.740
**DE2**				0.781	0.692
**DE3**				0.834	0.758

* Notes: Barlett’s test of sphericity is significant (p < 0.001),KMO value0.954(Greater than 0.70),Total variance explained65.347%(Greater than 50%),The variance for the first factor35.325%(Less than 50%).

As shown in Table 2, the rotated component matrix, which converged after five iterations using Kaiser normalization, displayed a clear four-factor structure: items WE1 to WE10 loaded onto Factor 1 (Website Design) with loadings between 0.750 and 0.792. This not only indicates that WE1 to WE10 are core characteristics of website design but also implies that website design can be considered a latent dimension. HV1 to HV7 loaded consistently onto Factor 2 (Hedonic Value) with loadings between 0.756 and 0.790; UV1 to UV7 loaded onto Factor 3 (Utilitarian Value) with loadings between 0.758 and 0.799; and DE1 to DE3 loaded onto Factor 4 (Decision) with loadings between 0.781 and 0.834. All factors showed no significant cross-loadings (factor loading difference > 0.20), further confirming the stability of the measurement tool’s multidimensional structural validity and discriminant validity under different experimental conditions. The rotated component matrix further indicated that all observed variables loaded above the basic requirement of 0.50 on their corresponding factors [145], with no significant cross-loadings. These results demonstrate that the model’s four latent factors were fully validated, and the scale can effectively represent these four latent factors, providing a solid foundation for subsequent data analysis.

For study 1, Cronbach’s alpha values for the dimensions related to internal consistency reliability ranged between 0.816 and 0.935, surpassing the recommended threshold of ≥0.70 [146]. S1 Table summarizes the factor loadings for all model structures, and Table 3 presents the CR and AVE values, indicating that both groups’ factor loadings ranged between 0.726 and 0.805, aligning with the recommendations of Fornell and Larcker that factor loadings should exceed 0.50 to achieve sufficient convergent validity. Moreover, the extracted AVE should exceed 0.50, and CR should be at least 0.70. All AVE values ranged between 0.589 and 0.597, while CR values varied between 0.816 and 0.935, indicating strong convergent validity [147–149].

**Table 3:** Confirmatory Factor Analysis in Study 1 and Study 2.

Item list	Study1:Gamification Degamification (778)			Study2:Gamification (477)		
	**AVE ≥ 0.5**	**CR ≥ 0.7**	**Cronbach’s a ≥ 0.7**	**AVE ≥ 0.5**	**CR ≥ 0.7**	**Cronbach’s a ≥ 0.7**
**Website Design**	0.591	0.935	0.935	0.600	0.938	0.937
**WD1 → WD10**						
**Hedonic Value**	0.590	0.909	0.910	0.594	0.911	0.911
**HV1 → HV7**						
**Utilitarian Value**	0.589	0.909	0.910	0.590	0.910	0.909
**UV1 → UV7**						
**Decision**	0.597	0.816	0.816	0.607	0.822	0.822
**DE1 → DE3**						
**Interpersonal value(Second-order Structure)**						
**SharingExperiences**				0.676	0.862	0.863
**SE1 → SE3**						
**Identity Recognition**				0.704	0.877	0.877
**ID1 → ID3**						
**Social Support**				0.656	0.851	0.850
**SS1 → SS3**						

* Notes: The goodness-of-fit statistics for the measurement model were χ²/df = 1.184, p < .001, RMSEA = 0.015, IFI = 0.995, TLI = 0.995, CFI = 0.995, SRMR = 0.0245, and GFI = 0.964, all indicating a good model-data fit.

Furthermore, we assessed discriminant validity by comparing the square root of the AVE values with the inter-construct correlations [147]. As shown in Table 4, the square root of each construct’s AVE (bold values on the diagonal) exceeds its correlations with all other constructs, providing strong empirical evidence for discriminant validity [150]. Additionally, the average variance extracted for each construct is greater than the squared correlations with other constructs, thereby confirming the adequacy of discriminant validity [151].

**Table 4:** Correlation Matrix in Study 1.

Construct	1	2	3	4
**Website**	**0.591**			
**Hedonic**	0.374	**0.590**		
**Utilitarian**	0.402	0.401	**0.589**	
**Decision**	0.394	0.426	0.392	**0.597**

* Notes: Note: All correlations were significant at p < 0.01.

#### 4.1.2 Structural model analysis and comparison.

This study employed multi-group structural SEM to test the cross-group applicability of the gamified and non-gamified models. The model fit analysis, as shown in Table 5, indicates that both the gamified group (χ²/df = 1.195, GFI = 0.932, CFI = 0.990, RMSEA = 0.022) and the non-gamified group (χ²/df = 1.256, GFI = 0.930, CFI = 0.986, RMSEA = 0.026) meet the fit standards recommended by (Hu and Bentler, 1999), with CFI > 0.95 and RMSEA < 0.06. This suggests that both baseline models exhibit stable measurement structures.

**Table 5:** Comparison of indirect effects models.

Model	χ²	df	χ²/df	GFI	AGFI	CFI	RMSEA	deltχ² (df)	p
**Gamification**	383.672	321	1.195	0.932	0.920	0.990	0.022		
**Degamification**	403.033	321	1.256	0.930	0.917	0.986	0.026		
**Unconstrained**	786.706	642	1.225	0.931	0.918	0.988	0.017		
**Measurement weights**	810.185	664	1.220	0.929	0.919	0.988	0.017	23.479 (22)	0.375
**Structural weights**	818.630	668	1.225	0.928	0.919	0.988	0.017	8.445 (4)	0.077

Based on the unconstrained model (χ²/df = 1.225, CFI = 0.988, RMSEA = 0.017), further measurement invariance testing revealed that constrained factor loadings across groups did not significantly worsen model fit (Δχ²(22) = 23.479, p = 0.375), confirming cross-group equivalence in latent variable factor loadings for both gamified and non-gamified groups. However, although the constrained structural weights model remained within acceptable fit standards (χ²/df = 1.220, CFI = 0.988, RMSEA = 0.017), a certain degree of chi-square difference was observed compared to the constrained measurement weights model (Δχ²(4) = 8.445, p = 0.077) [152]. This implies that there might be potential differences in the path coefficients of hedonic and utilitarian values of website personal value on decision intention under gamified and non-gamified conditions. Subsequent analysis may employ critical ratio (CR) tests to evaluate the extent to which different design modes moderate user decision-making mechanisms.

After conducting multi-group analysis and comparing gamified and non-gamified conditions, inter-group differences were found. The results showed that in the gamified group, as displayed in Table 6, the path coefficients for hedonic value (β = 0.463, p < 0.01) and utilitarian value (β = 0.431, p < 0.01) were significant, indicating that website design played a positive role in influencing personal hedonic and utilitarian values, thus supporting hypotheses H1 and H3.

**Table 6:** Path coefficient and hypothesis test results.

	Gamification	Degamification		
**Hypothesis**	**Coefficient (β)**	**Coefficient (β)**	**Critical ratios for difference**	**Hypothesis Results**
**H1,H2: Website design→Hedonic Value**	0.463***	0.310***	−1.638	H1:Supported, H2:Not Supported
**H3,H4: Website design→Utilitarian Value**	0.431***	0.397***	−0.827	H3:Supported, H4:Not Supported
**H5,H6: Utilitarian Value→Decision**	0.275***	0.290***	0.364	H5:Supported, H6:Not Supported
**H7,H8: Hedonic Value→Decision**	0.399***	0.255***	−2.109	H7:Supported, H8:Supported

*Note: significant level less than 0.05; *: significant level less than 0.01; ***. significant level less than 0.001.(CRs) ≥1.9 was significant.

**Fig 4:**
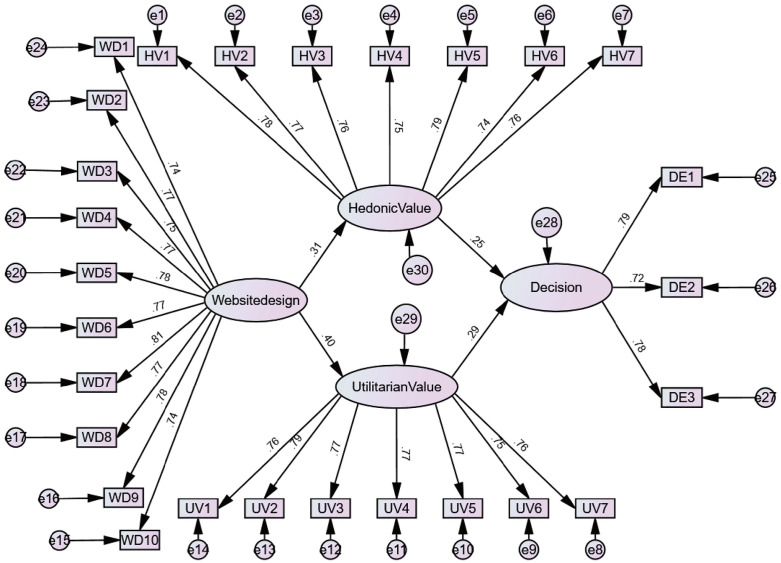
Gamified Path Coefficients.

**Fig 5:**
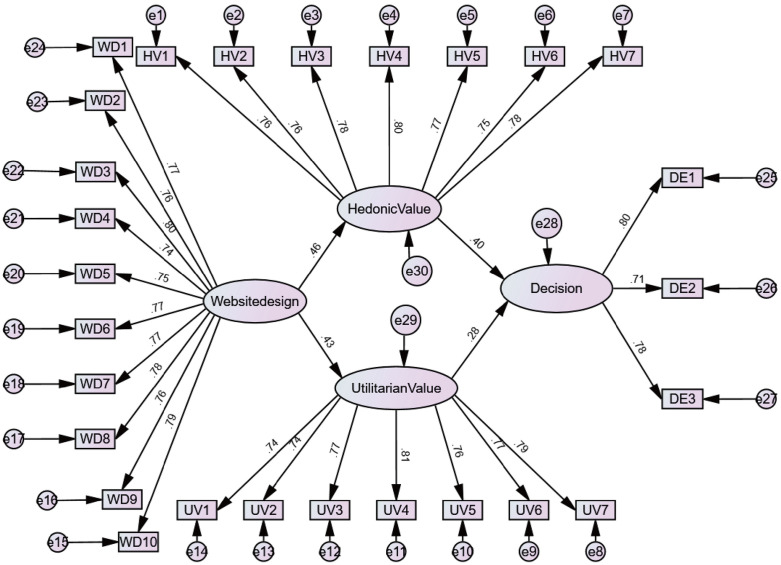
Non-Gamified Path Coefficients.

As shown in (Fig 4 and Fig 5), the control group (non-gamified design) also exhibited significant path coefficients for hedonic value (β = 0.310, p < 0.01) and utilitarian value (β = 0.397, p < 0.01), confirming its effectiveness as a reference group. Considering that a Critical Ratio (CR) ≥ 1.96 indicates a significant difference in parameters between groups [152], our observation of the impact of gamification on personal hedonic value experience (CR = −1.638) and utilitarian value experience (CR = −0.827) suggests that there is no significant difference between gamified and non-gamified conditions in terms of personal value experience. Therefore, hypotheses H2 and H4 are rejected. This necessitates an analysis in the discussion section of the website design content for both gamified and non-gamified approaches and the personal experiences generated, coupled with theoretical explanations.

Under gamified-driven design, the hedonic value (β = 0.399, p < 0.01) and utilitarian value (β = 0.275, p < 0.01) within personal value both show a positive influence on user decision-making, thereby supporting hypotheses H5 and H7. However, the control group results also indicate that personal value significantly influences the decision-making process, confirming their observability.

Regarding the influence of destination experience on decisions, gamification significantly enhanced the influence of users’ hedonic value on travel decisions compared to non-gamification (CR = −2.109, p < 0.05), thus confirming hypothesis H7. However, even though the utilitarian value brought by gamification has a positive and significant impact on decisions (0.275***), the non-gamified group showed a higher path coefficient (0.290***). The CR value between these two is 0.364, which led to the rejection of hypothesis H6. This makes it necessary for us to focus on analyzing the differences in destination information design between gamified and non-gamified websites in the discussion section, explaining the reason for the higher utilitarian value brought by non-gamified websites.

In the following sections, we will delve into analyzing the interpersonal value introduced by gamification and its influence on decisions through hedonic and utilitarian values, to determine whether it constitutes a key factor in decision-making.

### 4.2 Study 2 results analysis: The impact of gamification on decision-making through interpersonal value

#### 4.2.1 Reliability and validity of the measurement scale.

At the stage of study 2, since we had already conducted EFA for all factors except for the second-order structure in the previous phase, this stage focused on performing EFA on the first-order latent variables related to the three items of interpersonal value based on the sample size of study 2.

As summarized in Table 7, the KMO measure of sampling adequacy was 0.948, significantly exceeding the 0.70 threshold. The Bartlett’s test of sphericity yielded an approximate chi-square value of 10603.860 (df = 351, p < 0.001), meeting the strict criteria for data suitability. The common factor variance analysis showed that the extracted values for the three items ranged between 0.761 and 0.813, indicating high representativeness of each item within the latent factor structure. Principal component analysis (PCA) with varimax rotation extracted four components with eigenvalues greater than 1, with a cumulative variance explained rate of 69.349%.

**Table 7:** Exploratory Factor Analysis in Study 2.

Items	Component			Common Factor Variance
	1	2	3	
**IVSH1**		0.827		0.813
**IVSH2**		0.767		0.765
**IVSH3**		0.759		0.767
**IVID1**	0.818			0.814
**IVID2**	0.779			0.784
**IVID3**	0.815			0.810
**IVSU1**			0.774	0.761
**IVSU2**			0.758	0.772
**IVSU3**			0.760	0.778
**Barlett’s test of sphericity is significant**				p < 0.001
**KMO value(Greater than 0.70)**				0.948
**Total variance explained(Greater than 50%)**				69.349%
**The variance for the first factor(Less than 50%)**				34.837%

*Note: Because other factors have been analyzed in study 1, this table shows only second-order architecture factors.

For CFA, Table 3 summarizes the factor loadings, CR, and AVE values for all model structures. The AVE values ranged from 0.590 to 0.704, and the CR values ranged from 0.822 to 0.938, all meeting the required standards. The observed factor loadings were between 0.742 and 0.841, exceeding the 0.50 threshold, thus ensuring sufficient convergent validity. Furthermore, as shown in Table 8, the square roots of all AVE values were greater than the corresponding correlation coefficients, providing evidence for discriminant validity.

**Table 8:** Correlation Matrix in Study 2.

Construct	1	2	3	4	5	6	7	R²
**Website**	0.600							
**Decision**	0.451	0.607						
**Utilitarian**	0.384	0.388	0.590					
**Hedonic**	0.460	0.480	0.405	0.594				
**Sharing Experiences**	0.450	0.565	0.376	0.497	0.676			0.560
**Identity Recognition**	0.425	0.568	0.363	0.448	0.513	0.704		0.522
**Social Support**	0.480	0.579	0.393	0.500	0.603	0.602	0.656	0.645

Additionally, to ensure the validity of the second-order model, it is recommended that the R² values for each structural dimension should exceed 0.5 [135]. In this study, the R² values for the three items of interpersonal value—experience sharing, identity recognition, and social support—ranged from 0.522 to 0.645, demonstrating a significant impact on interpersonal value (p < 0.001). This confirms the feasibility of the second-order model.

#### 4.2.2 Structural model analysis and comparison.

This study systematically examined the path mechanism and theoretical structural rationality of gamified website design influencing decision intention by constructing first-order and second-order models. The results indicate that the composite higher-order representation of interpersonal value can significantly optimize model explanatory power. In the first-order model, as shown in Table 9, website design jointly influences decision intention by enhancing hedonic value (β = 0.466, p < 0.01), utilitarian value (β = 0.392, p < 0.01), and direct interpersonal value (β = 0.311, p < 0.01). The facilitating effects of hedonic and utilitarian value on interpersonal value were β = 0.378 and β = 0.207, respectively (both p < 0.01). Ultimately, the predictive strength of interpersonal value on decision reached β = 0.700 (p < 0.01). The overall model fit was acceptable (χ²/df = 2.375, RMSEA = 0.054, CFI = 0.920), although RMSEA was slightly outside the ideal range.

**Table 9:** Path coefficient and hypothesis test results.

	First-order model	second-order model	
Hypothesis	Coefficient (β)	Coefficient (β)	Hypothesis Results
**Website design→Hedonic Value**	0.466***	0.466***	
**Website design→Utilitarian Value**	0.392***	0.392***	
**H9: Website design→Interpersonal value**	0.311***	0.334***	Supported
**H10: Utilitarian Value→Interpersonal value**	0.207***	0.225***	Supported
**H11: Hedonic Value→Interpersonal value**	0.378***	0.405***	Supported
**H12: Interpersonal value→Decision**	0.700***	0.748***	Supported
**Model fit degree**			
**First-order model**	χ²/df = 2.375,χ² = 1420.309(df = 598), IFI = 0.920, TLI = 0.916, CFI = 0.920, RMSEA = 0.054 and SRMR = 0.0592		
**second-order model**	χ²/df = 1.148,χ² = 682.990 (df = 595), IFI = 0.991, TLI = 0.991, CFI = 0.991, RMSEA = 0.018 and SRMR = 0.0492		

*Note: significant level less than 0.05; *: significant level less than 0.01; ***. significant level less than 0.001.

**Fig 6:**
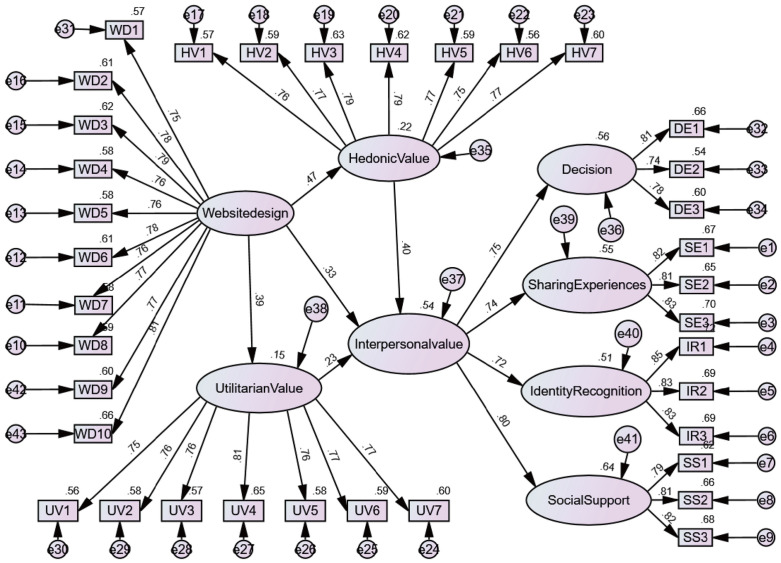
Second order model path coefficient.

Furthermore, the integrated model, which introduced interpersonal value as a second-order latent variable, not only maintained full path significance (e.g., website→second-order interpersonal value β = 0.466, p < 0.01; interpersonal value→decision intention β = 0.748, p < 0.01) but also showed significant effects of hedonic (β = 0.405, p < 0.01) and utilitarian value (β = 0.225, p < 0.01) on interpersonal value. In terms of model fit, the second-order model significantly improved model parsimony and fit (χ²/df = 1.148, RMSEA = 0.018, CFI = 0.991, SRMR decreased from 0.059 to 0.049), meeting the psychometric requirements for higher-order constructs. The empirical results demonstrate that both models can prove the influence of gamification on interpersonal value, thereby having a positive effect on people’s decisions, confirming that H9, H10, and H11, H12 are all supported. Among these, the dual advantages of the second-order model in parameter simplification and theoretical integration provided a more explanatory statistical analysis basis for verifying the hierarchical mechanism of interpersonal interaction value. Fig 6 allows for a more detailed discussion of the first-order factors of interpersonal value—experience sharing, identity recognition, and social support—and their influence paths on interpersonal value. All three showed significant effects, particularly the social support factor. Although they are not part of our hypothesized content, their path coefficients still indicate strong explanatory power and influenced the results.

## 5. Discussion

### 5.1 The influence of different gamification elements on decision-making pathways

Gamification has been widely studied for its ability to enhance hedonic value and utilitarian content in user experiences. For example, Shen et al. conducted an experiment incorporating gamification into destination-based knowledge interaction experiences using a Q-experiment design, demonstrating the significant role of gamification in providing tourists with hedonic experiences and destination-related utilitarian content [153]. Meanwhile, Hsu et al., in their study, utilized SEM to demonstrate that the two value experiences derived from gamification positively influence user attitudes and behavioral intentions, as indicated by significant path coefficient results [45,154]. However, prior research on gamification in destination websites has only fragmentedly explored the impact of website design on decision-making pathways and influencing factors—for example, the positive impact of website design on hedonic value or utilitarian value and the subsequent influence of these values on travel decisions [7,153]. Building on these studies, this paper identifies an effective way to integrate various variables influencing destination websites through gamification, establishing hedonic value and utilitarian value as key pathway variables in gamified website decision-making. Our results confirm the applicability and significance of a decision-making pathway model influenced by gamification, aligning with previous research findings.

According to this study’s findings, the standardized path coefficients for website design’s effect on hedonic value, utilitarian value, and utilitarian value’s effect on user decision-making did not show significant differences between the gamified and non-gamified groups (hypotheses H2 and H4 were both rejected). Since this experiment primarily considered website design (gamified versus non-gamified) and individual value experience, we need to explore this issue from these two perspectives. In terms of website design, our conclusion is inconsistent with Frías-Jamilena’s research, where gamification more positively influenced destination explanation compared to non-gamification [80]. In their experiment, they primarily adopted gamified elements such as badges, maps, challenges, and rewards. In our gamified design, we selected factors such as avatars, challenges, stories, and instant feedback as gamified content. The difference between the two lies in the trade-off between structural and content-based gamified elements [65]. Content-oriented gamification, such as game designs featuring avatars and stories, offers a stronger sense of immersion [155]. Nobre and others used the creation of co-creative gamified retail platforms to establish SEM models [156], demonstrating that these would bring players intense pleasure and engagement. Moreover, while playing, players would also glean desired information and understand the destination, ultimately leading tourists to develop stronger brand advocacy and influence decision orientation. This effectively explains how our conclusion shows that gamified websites brought positive effects to people’s hedonic and utilitarian value experiences and influenced their decisions (H1, H3, H5, H7, H8). However, these game experiences can also lead to a certain degree of cognitive load [157,158]. From the perspective of individual value, Turan verified that gamification strategies can enhance users’ learning motivation but also increase cognitive load, with utilitarian goals being the cause of this load. Clearly, gamified designs involve task objectives. For example, in our experimental process, users aiming to complete a certain objective might lead them to overlook understanding destination information through the gamified content itself. Furthermore, continuous challenges will reduce user patience, even if they can bring higher immersion [159,160]. This explains why, despite having built-in links to the non-gamified website in our gamified website design, the experimenters’ backend records showed that players did not click these links to access all the information. In contrast, our designed non-gamified website included images, videos, text, and user-friendly interactive components. These contents serve to provide utilitarian information and can very intuitively offer users an understanding of the destination [25]. In other words, the design advantage of non-gamified websites lies in their simple interaction and intuitive content. Based on the above discussion, these points well explain what led to the rejection of H2 and H4 in the conclusions. Not only that, we observed that while gamified websites showed a significant path coefficient in stimulating users’ utilitarian value (CR = −0.827), non-gamified websites exhibited a higher path coefficient in the influence of utilitarian value on decision-making (CR = 0.364). This indicates that, under the premise of consistent total information content across both types of websites, the difference may stem from varying methods of information presentation, which is closely related to the process design of gamified content. Poncin et al. noted in their research that even if gamification brings strong competitive advantages to smart storefronts, merely adding game mechanisms is insufficient to significantly increase tourists’ visitation intentions; achieving this depends on tourists’ proficiency in operating the game content to reach their goals [158,161]. This suggests that designs aimed at information comprehension should consider the user’s learning time cost. Furthermore, while making information fun through gamification, it can also transform this explicit information into a learning cost [99,160,162]. In our gamified experiment, the destination information presented was transient and could not be revisited for understanding and learning. In contrast, on non-gamified websites, users can repeatedly access information to help them make better decisions. Although gamification can effectively stimulate user interest and expose a large amount of information, this does not mean that users will repeatedly browse and convert this information into utilitarian value needed for decision-making, as user decision-making is a result of comprehensive consideration [93]. This perfectly explains why our hypothesis H6 was rejected.

In summary, even though we have demonstrated that gamification is significantly applicable to destination website decision pathways, after comparative experiments, we found that the application of different gamification elements can influence different path outcomes. For example, creating more immersive games based on website content can utilize elements such as avatars and stories. However, this might make the game time lengthy, bringing a stronger hedonic experience while potentially affecting user patience. Additionally, the linearity and transient nature of gamified information may not encourage users to repeatedly explore and thus help them make decisions. This finding will provide destination marketers with more detailed guidance on gamified design trends, enabling them to design game content that aligns with their destination’s development trends and desired effects, exploring the possibilities that gamification offers.

### 5.2 The impact of user profiles on interpersonal value

The empirical results of Study 2 indicate that gamified destination website design not only validates its role in driving hedonic value and utilitarian value within destination experiences but, more importantly, reveals how activating interpersonal value can positively influence tourists’ decision-making behavior. Previous research on whether gamification generates interpersonal value has not yet established mature models or frameworks. For example, in the study by Sigala et al., they categorized participants into different groups (Facebook social platform users and non-social platform users) to measure their experiences on gamified websites [163]. Their results showed that users who logged in through Facebook, a social platform, exhibited more active behaviors in content contribution and decision-making, aligning with our findings. However, their study did not classify user behaviors as an influencing factor, whereas our study effectively incorporates three dimensions of interpersonal value in social interactions—experience sharing, identity recognition, and social support—quantifying a previously population-based evaluation criterion. This newly developed model enhances reliability [164]. Furthermore, while their study predefined whether a group had social attributes before conducting the experiment, our study establishes a model framework that measures users’ post-experiment sharing behaviors and perceptions. This allows for a broader respondent base, as it does not limit measurement to a specific group.

However, user profiling remains a critical factor to consider. In the study by [165], low-income populations exhibited low responsiveness to utilitarian gamification elements, leading to an absence of observable interpersonal value—contradicting our study’s findings. To explain this discrepancy, we observed that respondents’ social platform usage habits and the number of friends they had were positively correlated with interpersonal value, a relationship also confirmed in the study [166]. For instance, if a respondent had an established habit of sharing, this sharing behavior would positively influence every variable in our decision-making pathway model (hedonic value, utilitarian value, and user decision-making) [167,168]. In contrast, users in Mantouka et al.’s study may have been less sensitive to the effects of gamification, leading to different experimental outcomes. This contradiction suggests that realizing interpersonal value in decision-making pathways requires the alignment of gamification design, user characteristics, and contextual attributes.

Moreover, according to user profiles, gamified content tends to be unique in its effects. Gamers and users engaged in gaming experiences develop specific behavioral habits [169]. Conducted cluster analysis based on user profiles, demonstrating that tourists at different destinations select gamified experiences based on personal preferences, thereby influencing outcome orientations [153]. For example, knowledge seekers prioritize stronger utilitarian value, while thrill-seekers and immersion-driven users pursue stronger hedonic value—both of which impact interpersonal value and decision outcomes. Additionally, effectively leveraging the interplay between gaming attributes and user profiles can significantly enhance the interpersonal value generated by gamification. As shown in the study [170], when players acquire cognitive content within a game, they are more likely to share it broadly within the gaming community. This insight helps explain why, if a user’s profile indicates that they are a sharing-oriented gamer, content dissemination becomes a key factor linking interpersonal value.

Therefore, user profiles not only influence the extent to which specific gamification designs achieve significant effects but also shape how users develop personal experiential habits before and during interactions with gamified products. These habits may, in turn, affect study outcomes. For example, if the surveyed users already exhibit strong sharing behaviors and habits, they are more likely to provide responses that favor interpersonal value data in subsequent experiments. This insight will guide future research in expanding and collecting behavioral data on different user gaming habits concerning specific decision-making pathways, as such factors may significantly influence study results.

### 5.3 Guiding implications for online destination website design

The findings of this study provide several guiding implications for the design of online destination websites: (1). When utilizing gamification as a website design framework, it is essential to consider the application of specific elements. Content-based elements (such as avatars and narratives) are better suited for scenarios where hedonic value dominates, as they enhance immersion. If the destination-based website has limited content, this approach can be used to extend the website’s hedonic appeal, thereby influencing user behavior. (2). From a utilitarian perspective, the integration of website and game content must not be excessively lengthy. If the goal is to convey website information effectively, small-scale games should be implemented. The duration of the experience and the level of challenge can impact user patience, potentially leading to negative effects. (3). Based on respondent demographic research, although this study has established a model for the impact of interpersonal value on decision-making pathways in destination experiences, tourists’ real-world conditions will affect their sensitivity to gamification. Notably, if tourists have extensive gaming experience, they may develop habitual gaming behaviors that influence decision-making outcomes. In future research, experimenters should take this factor into account when selecting respondents and consider it as a variable in explaining research findings.

### 5.4 Limitations and research directions

Although this study provides valuable insights into verifying the role of gamified design in user decision pathways and the construction of interpersonal value, several limitations remain. First, the current experiment did not conduct a stratified analysis of the differentiated impact of specific gamification elements on users’ hedonic and utilitarian values, thereby failing to explore the role attributes of different gamification elements in website design. Second, the experiment has not yet incorporated the measurement of users’ emotional responses. It is suggested that subsequent research introduce emotional variables into the design to prevent users from experiencing frustration or burnout due to challenging tasks, which could affect data representativeness and explanatory power. Additionally, the sample population primarily consisted of students and faculty from Chinese universities, presenting certain demographic limitations. Future research could further validate the model’s universality across different cultural backgrounds and diverse populations. Concurrently, the study of interpersonal value has not yet delved into the interactive feedback between users and their friends or family after information sharing. If we had designed more destination choices, these interactions might include positive or negative opinions, or trigger cognitive-level supplementary information processing, all of which could become important social mechanisms influencing users’ final decisions.

## 6. Conclusions

This study delves into the application potential of gamification mechanisms in tourism destination website design and establishes a comprehensive experimental model. We believe that the integration of gamification is expected to provide a generalizable new paradigm for destination website design. Furthermore, by incorporating “interpersonal value” into the analytical framework, this research significantly expands the theoretical possibilities regarding information dissemination pathways for destination websites.

Through two experiments, this study draws the following conclusions: (1) Compared to non-gamified designs, gamified destination websites can significantly enhance users’ hedonic and utilitarian values, and positively promote their intention to visit. (2) In the complete user decision-making path, gamified design did not demonstrate a significant advantage over non-gamified designs. Although both types of websites can provide significant utilitarian value, the path coefficient of utilitarian value to decision-making was lower in gamified websites. (3) The hedonic and utilitarian values stimulated by gamification have a significant positive impact on users’ perceived interpersonal value, which in turn indirectly promotes their travel decisions.

In summary, this research indicates that gamification is a design strategy that can be effectively integrated into destination websites to significantly enhance information dissemination. Moreover, this experience can activate the social attributes of destination websites, prompting users to consider interpersonal values more in their decision-making process, thereby expanding the pathways through which websites influence user behavior. We anticipate that future research can further deepen the detailed exploration of gamification elements and quantitatively analyze the impact of specific dimensions of interpersonal value on tourism decisions, with the aim of refining relevant theoretical models and providing more instructive practical recommendations.

## Supporting information

S1 List of measuring tool items. (ZIP)
