# Supplementary material for: Do the interpersonal effects of gamified online destination websites better stimulate tourists’ travel intentions?
Source: PLoS One. 2025 Oct 6;20(10):e0331397. doi: 10.1371/journal.pone.0331397 (PMC12500125; doi:10.1371/journal.pone.0331397)
Supplement: S1 — (ZIP) [file pone.0331397.s001.zip › Supporting information/S1_Table.pdf]

S1 Table. List of measuring tool items.

Item list	Loading		Document source
	Study1	Study2	
**Website Design**			
**WD1 The platform's design appears visually pleasant**	0.759	0.754	[1-5]
**WD2 The platform's design is aesthetically pleasing**	0.767	0.776	
**WD3 Using this platform improves my efficiency in finding destination-related content**	0.775	0.781	
**WD4 The platform makes it easier for me to accomplish tasks**	0.753	0.754	
**WD5 The interactive features of the platform meet my needs**	0.763	0.756	
**WD6 Interactions with the platform are efficient**	0.773	0.790	
**WD7 The platform's structure is clear and organized**	0.786	0.768	
**WD8 The platform provides clear and understandable results/feedback**	0.778	0.789	
**WD9 I trust the information found on this platform is reliable**	0.770	0.772	
**WD10 The platform appears trustworthy**	0.765	0.806	
**Hedonic Value**			
**HV1 The destination feels appealing**	0.769	0.756	[2, 6]
**HV2 The destination successfully stimulates my senses**	0.766	0.770	
**HV3 While browsing, I developed positive feelings toward the destination**	0.770	0.790	
**HV4 I can vividly imagine the enjoyment of visiting the destination**	0.773	0.788	
**HV5 I can clearly visualize the positioning and surroundings of each attraction at the destination**	0.781	0.768	
**HV6 While browsing, I can mentally map the location of each attraction in Gongshu District**	0.747	0.749	
**HV7 I believe accessing the destination would be challenging (Reverse-coded)**	0.772	0.772	
**Utilitarian Value**			
**UV1 I engaged in extensive thinking while using the website**	0.746	0.747	[6, 7]
**UV2 The website sparked my interest to learn more about the destination**	0.764	0.761	
**UV3 The website successfully stimulated my curiosity and problem-solving skills**	0.771	0.758	
**UV4 Through the website, I can clearly envision the experience of visiting the destination**	0.794	0.81	
**UV5 I believe there are many things to experience at the destination**	0.767	0.759	
**UV6 I find the website's description of the destination unrealistic (Reverse-coded)**	0.759	0.766	
**UV7 I believe it would be easy to interact with locals at the destination**	0.773	0.772	
**Decision**			
**DE1 The website/game was very useful in my travel decision-making**	0.805	0.815	[5, 8]

**DE2 The website/game significantly influenced my travel decisions**	0.726	0.742	
**DE3 If conditions permit, I plan to visit the destination in the near future**	0.786	0.779	
**Interpersonal value(Second-order Structure)**			
**SharingExperiences（First-order Structure）**			
**SE1 I share my experiences on social media with friends/family to express my interests and values**		0.818	[9, 10]
**SE2 Sharing my pre-trip decisions about the destination helps me better understand it through feedback**		0.814	
**SE3 Sharing my pre-trip decisions with friends/family strengthens our connection**		0.834	
**Identity Recognition（First-order Structure）**			
**IR1 I share destination-related content with friends/family/social media because they share similar interests**		0.841	[11, 12]
**IR2 When friends/family endorse my shared content, I feel more connected to them**		0.837	
**IR3 Their validation of my decisions from this platform enhances my sense of self-worth**		0.840	
**Social Support（First-order Structure）**			
**SS1 When planning trips, I seek advice from friends/family to reduce uncertainty**		0.772	[11, 12]
**SS2 Encouragement from friends/family increases my confidence in choosing a destination**		0.821	
**SS3 I am more likely to choose a destination if friends/family want to join me**		0.835	

Reference

1. Zhang H, Gordon S, Buhalis D, Ding X. Experience value cocreation on destination online platforms. Journal of Travel Research. 2018;57(8):1093-107.
2. Köchling A, Lohmann M. Assessing pre-travel online destination experience values of destination websites: scale development and validation. Information Technology & Tourism. 2022;24(4):457-84.
3. Pallud J, Straub DW. Effective website design for experience-influenced environments: The case of high culture museums. Information & Management. 2014;51(3):359-73.
4. Etemad-Sajadi R. The impact of online real-time interactivity on patronage intention: The use of avatars. Computers in human behavior. 2016;61:227-32.
5. Rahardja U, Aini Q, Graha YI, Tangkaw MR, editors. Gamification framework design of management education and development in industrial revolution 4.0. Journal of Physics: Conference Series; 2019: IOP Publishing.
6. Köchling A. Experiential marketing as a tool to enhance tourists' pre-travel online destination experiences? A web-based experiment. Journal of Destination Marketing & Management. 2021;22:100669.
7. Bae J-H, Jeon H-M. Exploring the relationships among brand experience, perceived product quality, hedonic value, utilitarian value, and brand loyalty in unmanned coffee shops during the COVID-19 pandemic. Sustainability. 2022;14(18):11713.

8. Erul E, Woosnam KM, Salazar J, Uslu A, Santos JAC, Sthapit E. Future travel intentions in light of risk and uncertainty: An extended theory of planned behavior. Sustainability. 2023;15(22):15729.
9. Kim AK, Brown G. Understanding the relationships between perceived travel experiences, overall satisfaction, and destination loyalty. Anatolia. 2012;23(3):328-47.
10. Kang M, Schuett MA. Determinants of sharing travel experiences in social media. Journal of Travel & Tourism Marketing. 2013;30(1-2):93-107.
11. Sedera D, Lokuge S, Atapattu M, Gretzel U. Likes—The key to my happiness: The moderating effect of social influence on travel experience. Information & Management. 2017;54(6):825-36.
12. Lemay Jr EP, Ryan JE, Teneva N. Pursuing interpersonal value: An interdependence perspective. Journal of Personality and Social Psychology. 2021;120(3):716.
