# Supplementary figures and images for: Do the interpersonal effects of gamified online destination websites better stimulate tourists’ travel intentions?
Source: PLoS One. 2025 Oct 6;20(10):e0331397. doi: 10.1371/journal.pone.0331397 (PMC12500125; doi:10.1371/journal.pone.0331397)

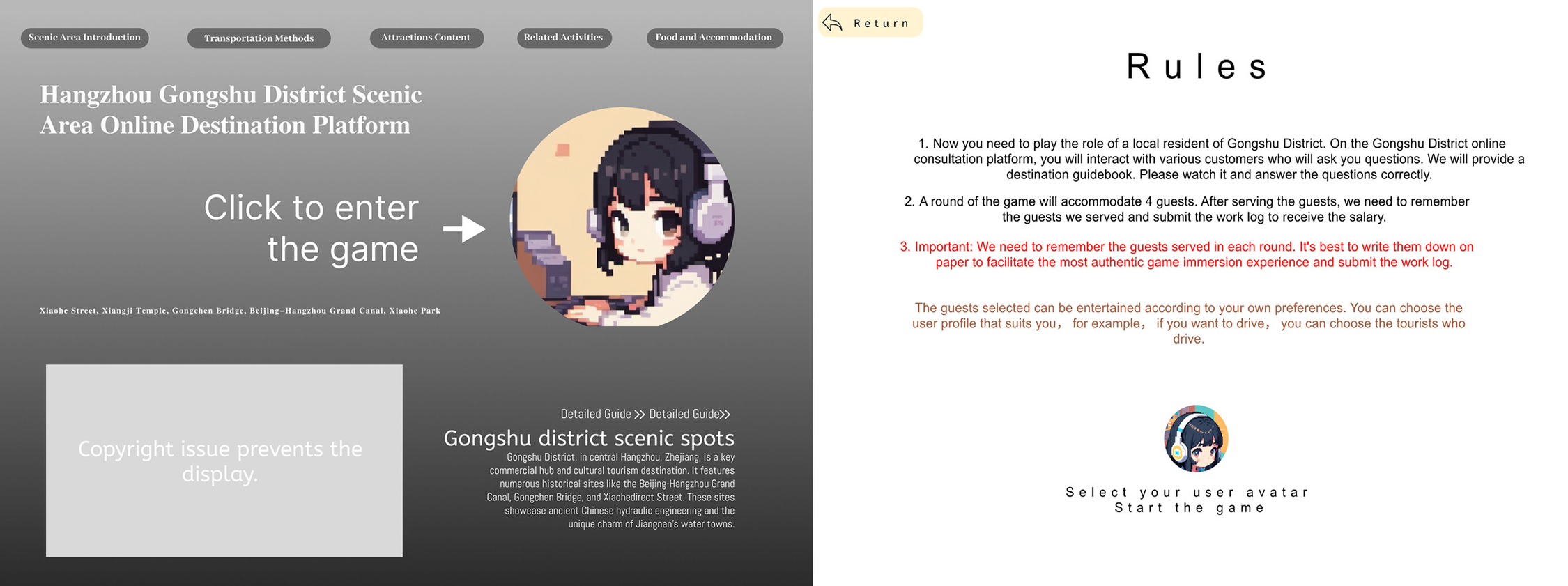

Supplement: S1 — (ZIP) [file pone.0331397.s001.zip › Supporting information/S2_Fig.tif]

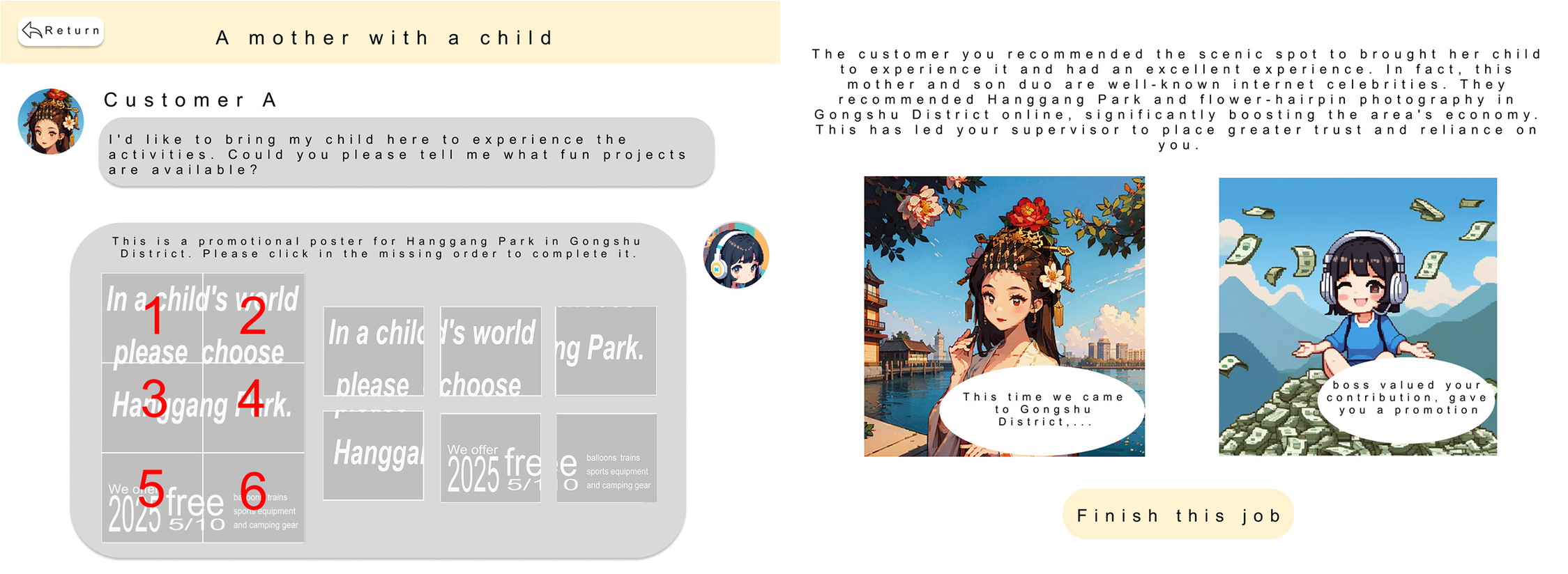

Supplement: S1 — (ZIP) [file pone.0331397.s001.zip › Supporting information/S3_Fig.tif]

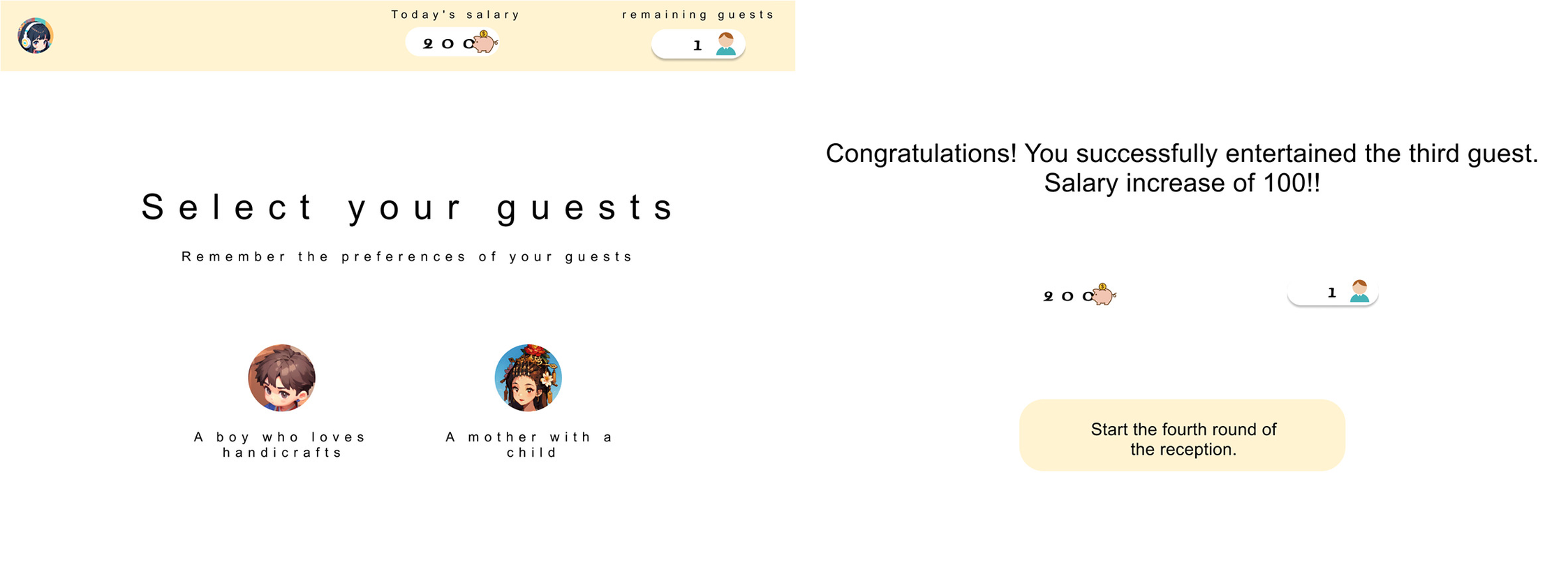

Supplement: S1 — (ZIP) [file pone.0331397.s001.zip › Supporting information/S4_Fig.tif]

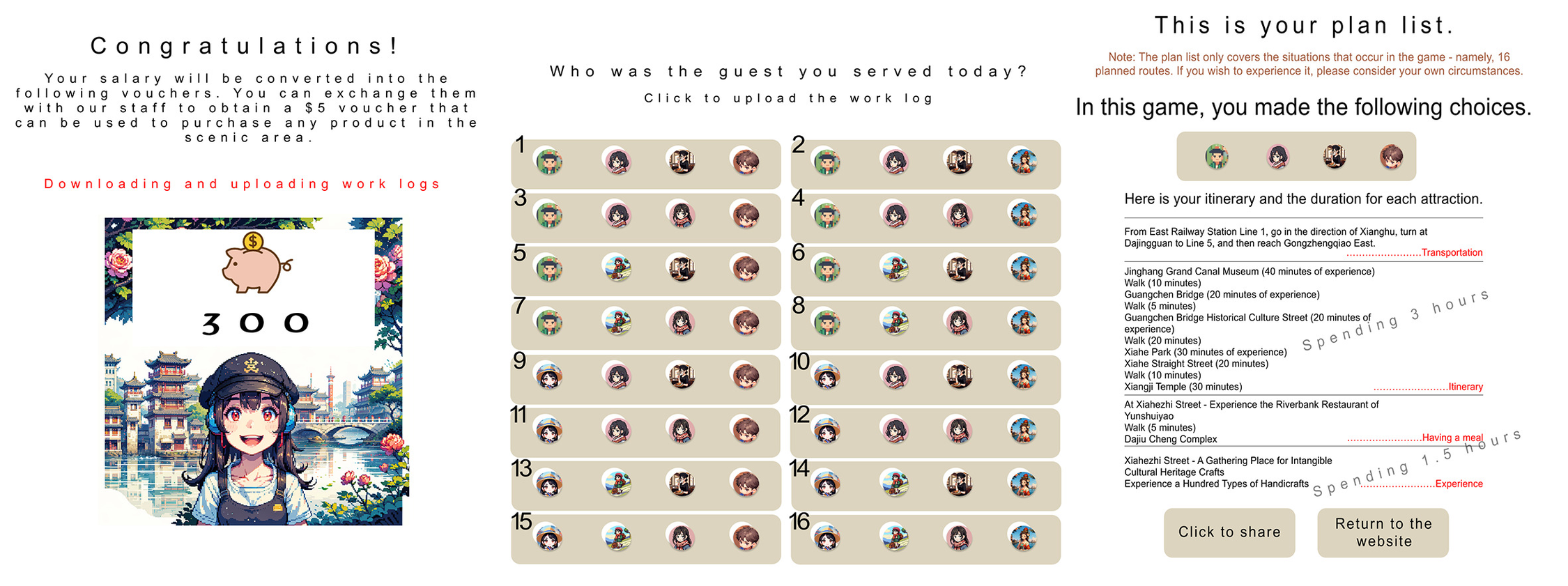

Supplement: S1 — (ZIP) [file pone.0331397.s001.zip › Supporting information/S5_Fig.tif]
